# Agricultural residues for cellulolytic enzyme production by *Aspergillus niger*: effects of pretreatment

**DOI:** 10.1007/s13205-015-0294-5

**Published:** 2015-03-27

**Authors:** Aliyu Salihu, Olagunju Abbas, Abdullahi Balarabe Sallau, Md. Zahangir Alam

**Affiliations:** 1 Department of Biochemistry, Ahmadu Bello University Zaria, Zaria, Nigeria; 2 Department of Biotechnology Engineering, Faculty of Engineering, Bioenvironmental Engineering Research Centre (BERC), International Islamic University Malaysia (IIUM), Gombak, 50728 Kuala Lumpur, Malaysia

**Keywords:** Agricultural residues, *Aspergillus niger*, Cellulase, Pretreatment

## Abstract

Different agricultural residues were considered in this study for their ability to support cellulolytic enzyme production by *Aspergillus niger*. A total of eleven agricultural residues including finger millet hulls, sorghum hulls, soybean hulls, groundnut husk, banana peels, corn stalk, cassava peels, sugarcane bagasse, saw dust, rice straw and sheanut cake were subjected to three pretreatment (acid, alkali and oxidative) methods. All the residues supported the growth and production of cellulases by *A. niger* after 96 h of incubation. Maximum cellulase production was found in alkali-treated soybean hulls with CMCase, FPase and β-glucosidase yields of 9.91 ± 0.04, 6.20 ± 0.13 and 5.69 ± 0.29 U/g, respectively. Further studies in assessing the potential of soybean hulls are being considered to optimize the medium composition and process parameters for enhanced cellulase production.

## Introduction

Agricultural residues represent vast raw materials that can be utilized for production of value-added products. The major components of these raw materials, including cellulose (35–50 %), hemicellulose (20–35 %), lignin (15–25 %) and a number of other compounds make up the residues (Wyman 1994).

Thus, cellulose being the most abundant polysaccharide constituents of agricultural residues consists of β-1,4 linear polymers of 8000–12,000 glucose units. It is mostly found in crystalline, water-insoluble form, and cannot be easily hydrolyzed by most microorganisms (Saha 2004; Clarke 1997).

Some of the microorganisms that have been reported to hydrolyze this insoluble polymer into soluble monomeric units include *Bacillus pumilus* EB3 (Ariffin et al. 2008), *Pseudomonas* sp. WLUN024 (Xu et al. 2005), *Trichoderma reesei* (Wen et al. 2005), *Trichoderma harzianum* (Alam et al. 2009), *Penicillium echinulatum* (Sehnem et al. 2006), *Aspergillus Phoenicis* (Wen et al. 2005)*, Phanerochaete chrysosporium* (Khan et al. 2007) and *Aspergillus niger* MS82 (Sohail et al. 2009); all of which require the action of cellulases. Cellulase is a multi-component enzyme system that works synergistically in hydrolyzing the cellulosic substrates to glucose. Mostly three enzymes are involved; endo-β-d-glucanase (EC 3.2.1.4) which catalyzes the random hydrolysis of soluble and insoluble cellulose chains. Exo-β-d-glucanase (EC 3.2.1.19) aids in releasing cellobiose from reducing and non-reducing ends of cellulose, and hydrolysis of cellobiose to glucose is carried out by β-glucosidase (EC 3.2.1.37) (Bhat and Bhat 1997; Bhat 2000; Sohail et al. 2009).

Cellulases have been used in a number of industrial processes. The most notable applications are in textile, paper and pulp, food and animal feed, fuel and chemical industry, waste management, and pharmaceutical industry (Bhat 2000; Bhat and Bhat 1997).

Thus, concerted efforts are being made by researchers in exploring different lignocellulosic residues for cellulase production using different fermentation techniques. Some of the agricultural residues reported for cellulase production include apple pomace (Dhillon et al. 2012), Coir pith (Jabasingh 2011), oil palm empty fruit bunches (Alam et al. 2009), rice straw (Khan et al. 2007), wheat bran (Xu et al. 2005), switchgrass, corn stover (Zhang et al. 2012), sugarcane bagasse (Maeda et al. 2013), grape pomace (Diaz et al. 2012), rice bran (Liu et al. 2011), corn cobs, rice husk, saw dust and wheat straw (Bansal et al. 2012).

The use of these cheaply available residues contributes to reducing the total enzyme production cost, as raw materials account for up to 40–60 % of the production cost (Wen et al. 2005). Based on this, there is still need for exploring various agricultural residues in order to come up with an effective fermentation medium for cellulase production for wider industrial application.

Additionally, efficient utilization of agricultural residues in bioconversion processes may require different pretreatment techniques, which aid in breaking the lignin seal and distort the organizational structure of complex organic components. Thus, pretreatment of any agricultural residues as described by Sun and Cheng (2002) should not cause degradation of carbohydrate or development of any byproducts that could be inhibitory to the subsequent experimental processes involving hydrolysis and fermentation. Also, improving the formation of sugar hydrolysates by reducing cellulose crystallinity and increasing the porosity of agricultural residues are to be prioritized.

Chemical pretreatment methods have been used in enhancing the bioconversion of lignocellulosic residues for cellulase production. Alkaline pretreatment being the most effective chemical method results in delignification of agricultural residues which leads to breakage of ester bonds cross-linking lignin and xylan, and in effect aids in increasing the porosity of biomass (Sun and Cheng 2002). In case of acid pretreatment, particle size, temperature, reaction time and acid concentration as well as liquid-to-solid ratio are the major factors affecting the process (Zhao et al. 2008). While using H_2_O_2_ aids in delignification of biomass via oxidative reactions to detach and solubilize the lignin by weakening the lignocellulosic matrix, this contributes to improving enzyme digestibility (Silverstein et al. 2007).

The present study was aimed at determining the best substrate (from different agricultural residues) as well as the pretreatment method for cellulolytic enzyme production using *A. niger*. This is because among the cellulase producing microorganisms, *A. niger* has been reported to be efficient in the synthesis of all the three cellulolytic enzymes (Sohail et al. 2009). The information obtained in this study would be helpful in developing a cost-effective process for cellulase production.

## Materials and methods

### Microorganism

*A. niger* used in this study was obtained from culture collection of Department of Crop Protection, Ahmadu Bello University Zaria and maintained on potato dextrose agar slants.

### Agricultural residues and pretreatments

Eleven agricultural residues available in Zaria were collected locally; these include finger millet hulls, sorghum hulls, soybean hulls, groundnut husk, banana peels, corn stalk, cassava peels, sugarcane bagasse, saw dust, rice straw and sheanut cake. The residues were oven-dried in a hot-air oven (60 °C), before being ground and sieved to 1 mm particle size. The samples were then stored under appropriate conditions in plastic containers until further use. Characterization of these residues was carried out based on sequential fractionation method of Datta (1981) as modified by Arora and Sharma (2009), and the results in terms of cellulose, hemicellulose and lignin were indicated in Table 1.

**Table 1:** Composition of agricultural residues used in this study

S/no.	Substrate	Cellulose (%)	Hemicellulose (%)	Lignin (%)
1	Finger millet hulls	25 ± 0.73	32 ± 1.38	4 ± 1.66
2	Sorghum hulls	39 ± 0.82	35 ± 0.07	4 ± 0.11
3	Banana peels	11 ± 1.12	9 ± 0.08	3 ± 0.55
4	Corn stalk	30 ± 1.19	33 ± 2.63	15 ± 0.67
5	Cassava peels	39 ± 0.34	25 ± 0.41	8 ± 0.52
6	Saw dust	44 ± 1.77	17 ± 0.14	21 ± 1.92
7	Sheanut cake	26 ± 0.26	9 ± 0.33	21 ± 0.09
8	Rice straw	35 ± 1.31	27 ± 1.97	16 ± 2.01
9	Soybean hulls	35 ± 0.12	16 ± 0.21	4 ± 0.19
10	Sugarcane bagasse	45 ± 0.52	26 ± 0.34	19 ± 0.13
11	Groundnut husk	36 ± 1.41	20 ± 0.88	25 ± 1.03

Values are means ± standard deviations

Acid, alkali and hydrogen peroxide pretreatments of the samples were carried at solid loading of 10 % (w/v) using 1 N H_2_SO_4_ and 1 N NaOH and 1 N H_2_O_2_, respectively. The samples were autoclaved for 20 min at 121 °C temperature and 15 psi pressure based on the method described by Singh and Bishnoi (2013) with slight modification. The samples were washed repeatedly with water to remove the solvents used for pretreatment and then dried at 60 °C to a constant weight.

### Cellulase production under solid state fermentation

The target in this study is to select the best substrate as well as pretreatment method for the enzyme production; as such, the substrates were considered with no exogenous addition of any nutrient. Cellulase production experiments were carried in 250-ml Erlenmeyer flask as described by Bansal et al. (2012) containing 5 g each of the substrate (untreated or treated with acid, alkali and peroxide) of 1 mm particle size and moistened with distilled water in a ratio of 1:1.5. The initial pH of each set up was fixed at 6.5 ± 0.1 by adding few drops of 1 N NaOH, and the fermentation was carried out under uncontrolled pH condition.

Following the autoclaving of the flasks at 121 °C (15 psi) for 20 min, 7 mm cut of PDA containing actively growing colonies of 72-h-old culture of *A. niger* was aseptically added to each flask and incubated at 30 °C for 96 h as reported by Bansal et al. (2012). After the fermentation, 50 ml of sterile distilled water was added to each flask and shaken on a rotary shaker (180 rpm) for 1 h. The mixture was centrifuged at 5000×*g* for 10 min, and the supernatant was used to assay for the enzyme activity.

### Determination of enzyme assays

The components of the cellulase system which include carboxymethyl cellulase (CMCase), filter paper activity (FPase) and β-glucosidase were determined based on the methods described by Ghose (1987) and Mandels et al. (1976). One unit (U) of the enzyme was defined as the amount of enzyme that releases 1 µmol of reducing sugar per minute from CMC, Whatman filter paper and salicin under standard assay conditions of 0.05 M acetate buffer, pH 4.8 at 50 °C using dinitrosalicylic acid reagent. The results are expressed in terms of unit per gram of solid substrate (U/g).

## Results and discussion

Agricultural residues are generated in large quantities in many countries; most of which are underutilized and considered as waste especially in developing countries. Significant efforts have been made by several researchers in converting these residues to valuable products including biofuels, animal feed, biofertilizer, and enzymes. (Dashtban et al. 2009; Sánchez 2009). These processes help in controlling some of the environmental challenges associated with their disposal. The polymeric constituents of agricultural residues used in this study were determined in terms of cellulose, hemicellulose and lignin as presented in Table 1. This characterization is important in identifying the components that can support the growth of microorganisms for value-added product formation.

The cellulolytic activities in terms of CMCase, FPase and β-glucosidase obtained in this study were presented in Tables 2, 3, 4 and 5. Interestingly, cellulolytic enzyme activities were recorded in unpretreated residues (Table 2); this indicates that reduction in particle size by milling to 1 mm can be considered as the first step of pretreatment because it aids in increasing the substrate’s surface area and decreasing cellulose crystallinity which make the available carbon sources accessible for microbial utilization (Kumar et al. 2009). In addition, *A. niger* is a versatile organism with strong ability to utilize the available nutrients present in different agricultural residues and to produce several enzyme systems during the bioconversion process. A clear trend was seen in all the three cellulolytic enzyme systems (CMCase, FPase and β-glucosidase), where their lowest activities were found in sheanut cake. However, the values were not statistically different from those of finger millet hulls, sorghum hulls and corn stalk. Rice straw and sugarcane bagasse showed the highest activities of 1.76 ± 0.08, 1.22 ± 0.03, 0.91 ± 0.04 and 1.66 ± 0.19, 1.01 ± 0.07, 0.84 ± 0.01 U/g in terms of CMCase, FPase and β-glucosidase, respectively.

**Table 2:** Production of cellulase by *A. niger* using different agricultural residues without pretreatment

S/no.	Substrate (untreated)	CMCase (U/g)	FPase (U/g)	β-glucosidase (U/g)
1	Finger millet hulls	0.31 ± 0.10^a,b^	0.25 ± 0.10^a,b^	0.14 ± 0.02^a^
2	Sorghum hulls	0.45 ± 0.09^a,b^	0.39 ± 0.09^b,c^	0.21 ± 0.03^a^
3	Banana peels	0.25 ± 0.08^a^	0.31 ± 0.08^a,b,c^	0.67 ± 0.05^c^
4	Corn stalk	0.71 ± 0.10^a,b^	0.44 ± 0.04^c^	0.79 ± 0.09^d,e^
5	Cassava peels	1.30 ± 0.08^c,d,e^	0.78 ± 0.07^d^	0.59 ± 0.04^c^
6	Saw dust	0.72 ± 0.10^b^	0.47 ± 0.02^c^	0.40 ± 0.03^b^
7	Sheanut cake	0.09 ± 0.01^a,b^	0.19 ± 0.02^a^	0.09 ± 0.01^a^
8	Rice straw	1.76 ± 0.08^e^	1.22 ± 0.03^f^	0.91 ± 0.04^e^
9	Soybean hulls	1.29 ± 0.14^c,d^	0.99 ± 0.04^e^	0.81 ± 0.01^e^
10	Sugarcane bagasse	1.66 ± 0.19^d,e^	1.01 ± 0.07^e^	0.84 ± 0.01^e^
11	Groundnut husk	1.19 ± 0.06^c^	0.98 ± 0.01^e^	0.84 ± 0.08^e^

Values are means ± standard deviations. Data with the same superscript letter were not significantly different (*p* < 0.05; Tukey test)

**Table 3:** Production of cellulase by *A. niger* using acid-treated agricultural residues

S/no.	Substrate (acid treated)	CMCase (U/g)	FPase (U/g)	β-glucosidase (U/g)
1	Finger millet hulls	0.80 ± 0.20^a,b^	0.90 ± 0.02^a,b^	0.31 ± 0.05^a,b^
2	Sorghum hulls	0.96 ± 0.04^b^	0.99 ± 0.07^b^	0.46 ± 0.01^a^
3	Banana peels	0.50 ± 0.03^a,c^	0.71 ± 0.01^a^	1.01 ± 0.08^c^
4	Corn stalk	1.08 ± 0.10^b^	0.83 ± 0.05^a,b^	1.09 ± 0.10^c,d^
5	Cassava peels	2.30 ± 0.30^d^	1.70 ± 0.13^c,d^	1.27 ± 0.11^d^
6	Saw dust	1.10 ± 0.20^b^	0.98 ± 0.02^b^	0.72 ± 0.05^e^
7	Sheanut cake	0.31 ± 0.01^c^	0.25 ± 0.12^e^	0.13 ± 0.01^b^
8	Rice straw	3.20 ± 0.06^e^	2.10 ± 0.08^g^	2.00 ± 0.14^f^
9	Soybean hulls	3.12 ± 0.14^e^	2.05 ± 0.07^f,g^	1.99 ± 0.01^f^
10	Sugarcane bagasse	3.00 ± 0.10^e^	1.85 ± 0.05^d,f^	1.92 ± 0.03^f^
11	Groundnut husk	2.87 ± 0.26^e^	1.61 ± 0.14^c^	1.96 ± 0.11^f^

Values are means ± standard deviations. Data with the same superscript letter were not significantly different (*p* < 0.05; Tukey test)

**Table 4:** Production of cellulase by *A. niger* using oxidative treatment of agricultural residues

S/no.	Substrate (H_2_O_2_ treated)	CMCase (U/g)	FPase (U/g)	β-glucosidase (U/g)
1	Finger millet hulls	1.43 ± 0.16^a,b^	0.91 ± 0.01^a^	1.10 ± 0.15^a,b,c^
2	Sorghum hulls	1.55 ± 0.02^a,b^	1.00 ± 0.03^a^	0.77 ± 0.20^a,b^
3	Banana peels	1.79 ± 0.13^a,b^	1.10 ± 0.22^a^	1.01 ± 0.10^a,b,c^
4	Corn stalk	2.01 ± 0.02^a^	1.32 ± 0.01^a,b^	1.27 ± 0.10^c^
5	Cassava peels	3.04 ± 0.07^c^	1.48 ± 0.11^a,b^	1.25 ± 0.03^b,c^
6	Saw dust	1.65 ± 0.02^a,b^	1.47 ± 0.06^a,b^	1.03 ± 0.03^a,b,c^
7	Sheanut cake	1.20 ± 0.30^b^	0.64 ± 0.04^a^	0.68 ± 0.10^a^
8	Rice straw	5.30 ± 0.40^d^	3.60 ± 0.33^c^	2.31 ± 0.14^d^
9	Soybean hulls	5.65 ± 0.33^d^	3.58 ± 0.11^c^	3.16 ± 0.36^e^
10	Sugarcane bagasse	4.61 ± 0.23^e^	2.93 ± 0.65^c,d^	2.22 ± 0.24^d^
11	Groundnut husk	4.43 ± 0.15^e^	2.39 ± 0.95^d^	2.04 ± 0.07^d^

Values are means ± standard deviations. Data with the same superscript letter were not significantly different (*p* < 0.05; Tukey test)

**Table 5:** Production of cellulase by *A. niger* using alkali-treated agricultural residues

S/no.	Substrate (alkali treated)	CMCase (U/g)	FPase (U/g)	β-glucosidase (U/g)
1	Finger millet hulls	1.63 ± 0.10^a,b^	1.05 ± 0.10^a^	1.30 ± 0.04^a,b^
2	Sorghum hulls	1.64 ± 0.08^a,b^	1.11 ± 0.20^a^	0.99 ± 0.05^a^
3	Banana peels	2.00 ± 0.02^a^	1.96 ± 0.10^b^	1.22 ± 0.01^a,b^
4	Corn stalk	3.30 ± 0.15^c^	2.74 ± 0.09^c^	2.09 ± 0.01^c^
5	Cassava peels	4.10 ± 0.01^d^	3.03 ± 0.04^c,d^	2.93 ± 0.11^d^
6	Saw dust	3.04 ± 0.12^c^	1.81 ± 0.06^b^	1.77 ± 0.10^b,c^
7	Sheanut cake	1.34 ± 0.03^b^	0.89 ± 0.10^a^	0.87 ± 0.02^a^
8	Rice straw	8.81 ± 0.21^e^	5.23 ± 0.19^e^	4.10 ± 0.62^e^
9	Soybean hulls	9.91 ± 0.04^f^	6.20 ± 0.13^f^	5.69 ± 0.29^f^
10	Sugarcane bagasse	8.33 ± 0.50^e^	5.06 ± 0.06^e^	4.09 ± 0.44^e^
11	Groundnut husk	5.88 ± 0.16^g^	3.08 ± 0.09^d^	3.97 ± 0.01^e^

Values are means ± standard deviations. Data with the same superscript letter were not significantly different (*p* < 0.05; Tukey test)

Acid pretreatment (Table 3) yielded higher cellulolytic enzyme production than the unpretreated substrates (Table 2), and several disadvantages have been linked with its utilization including formation of degradation products of pentoses, hexoses and other compounds, such as furfural, hydroxyl-methyl furfural, acetic acid, formic acid, levulinic acid, which affect microbial bioconversion processes (Balat et al. 2008). Thus, soybean hulls appeared to have higher percentage improvement in cellulase production with 58, 51 and 59 % (equivalent to 3.12 ± 0.14, 2.05 ± 0.07 and 1.99 ± 0.01 U/g) for CMCase, FPase and β-glucosidase, respectively, when compared with the unpretreated substrates. Other residues with improved cellulolytic activities following the acid pretreatment include rice straw, sugarcane bagasse and groundnut husk.

In case of oxidative pretreatment using H_2_O_2_, increment in cellulase production was observed in all the residues (Table 4), and maximum cellulase production was found in soybean hulls with 5.63 ± 0.33 U/g for CMCase, 3.58 ± 0.11 U/g for FPase and 3.16 ± 0.36 U/g β-glucosidase followed by rice straw with 5.30 ± 0.40, 3.60 ± 0.33 and 2.31 ± 0.14 U/g for CMCase, FPase and β-glucosidase, respectively. Sheanut cake had the least cellulase production; this is because of its compositions (especially the residual oil content) as it was reported to be a potential substrate for lipase production by *A. niger* (Salihu et al. 2013). Also, Hon and Shiraishi (2001) noted that several reactions may occur during oxidative pretreatment which include displacement of side chains, electrophilic substitution and cleavage of alkyl aryl ether linkages or oxidative cleavage of aromatic nuclei. These reactions help in removing the hemicellulose and lignin by increasing the accessibility of the cellulose (Singh and Bishnoi 2013). However, the presence of these hydrolysates may cause some inhibitory effects that result in lower activities compared to alkaline pretreatment method.

Based on all the results, alkaline pretreated residues (Table 5) showed the highest cellulolytic enzyme production compared to the unpretreated, acid- and H_2_O_2_-treated residues. This could be related to minimum amount of lignin content and increased internal surface area as well as fiber distension in the alkali-treated residues (Sun and Cheng 2002). Thus, the available cellulose and hemicellulose following this pretreatment favor microbial growth and enzyme production.

Maximum cellulolytic enzyme production was observed in alkali-treated soybean hulls of 9.91 ± 0.04, 6.20 ± 0.13 and 5.69 ± 0.29 U/g in terms of CMCase, FPase and β-glucosidase, respectively. Generally, soybean hulls were reported to consist of cellulose (46–51 %), hemicellulose (16–18 %) and lignin (1.4–2 %), making them to be a good source of fermentable sugars for utilization in formation of several value-added products (Corredor et al. 2008). However, the composition of soybean hulls used in this study was lower in terms of cellulose (35 ± 0.12 %) and higher in lignin content (4 ± 0.19 %). The hemicellulose was found to be within the range (16 ± 0.21 %) as indicated in Table 1.

Most studies where higher cellulase production was reported involved the use of additional medium components to enhance the production. Maximum FPase and β-glucosidase activities of 133.68 ± 5.44 and 60.09 ± 3.43 U/g, respectively, were obtained by *A. niger* NRRL-567 when apple pomace was supplemented with CuSO_4_ and veratryl alcohol. However, supplementation of apple pomace with lactose resulted in better CMCase activity of 172.31 ± 14.21 U/g (Dhillon et al. 2012)

The findings in this study agree with what were reported by Zhang et al. (2012) using acid and alkali-treated samples of switch grass, corn stover and anaerobically digested manure fiber. The three alkali-treated samples gave higher cellulase activities than acid-treated ones. Also, results obtained when using alkali-treated coir pith, a by-product of coir fiber extraction process for cellulase production by *A. nidulans* showed maximum CMCase, FPase and cellobiase activities of 28.64, 10.23 and 4.31 U/g, respectively (Jabasingh 2011).

**Fig. 1:**
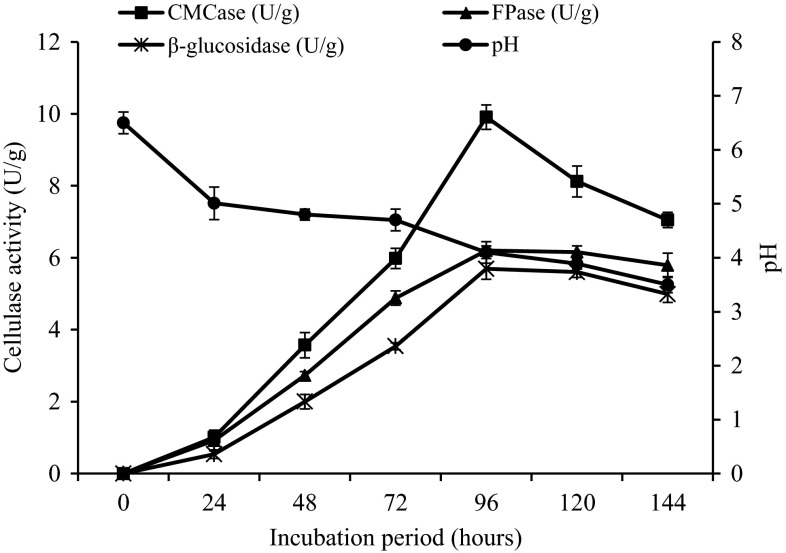
Cellulase production in alkali-treated soybean hulls based on initial pH of 6.5 at different fermentation time

Effect of incubation period on alkali-treated soybean hulls as the most promising residues with higher cellulolytic enzyme activities was studied, and maximum production was observed after 96 h (Fig. 1). Further increase in incubation period resulted in decreased enzyme activities. Thus, incubation period of 96 h was found to be sufficient for maximum cellulase production in alkali-treated soybean hulls.

Similarly, pH of the fermentation medium decreased from the initial pH of 6.5 to 4.1 ± 0.2 (at 96 h) when the cellulase production was at its optimum. The pH decreased further to 3.5 ± 0.15 at the end of the experiment (144 h) as indicated in Fig. 1. The decrease in pH could be related to the formation of acidic metabolite during the bioconversion process, and the final pH of 3.5 may still be within the favorable condition for efficient fungal growth as suggested by Molla et al. (2004). Similar trend in pH reduction was also reported by Alam et al. (2009) during cellulase production by *T. harzianum* T2008 using oil palm empty fruit bunches as substrate.

Further studies in optimizing the medium composition as well as process parameters for enhanced cellulase production using soybean hulls as novel substrate are currently being considered.

## Conclusion

Eleven agricultural residues were considered for cellulolytic enzyme production using three pretreatment methods (acid, oxidative and alkaline). Alkaline pretreatment was found to be the most efficient method with higher enzyme production. Soybean hulls showed higher activities in terms of CMCase, FPase and β-glucosidase after 96 h of incubation. Using this cheap and renewable residue for cellulolytic enzyme production by *A. niger* boosts its economic value which is not comparable with its current use as animal feed.
